# Melanin Bias in Cerebral fNIRS: A Monte Carlo Study Toward Equitable Optical Brain Sensing

**DOI:** 10.3390/bios16100538

**Published:** 2026-09-25

**Authors:** Murad Althobaiti

**Affiliations:** Department of Biomedical Engineering, College of Engineering, Imam Abdulrahman Bin Faisal University, P.O. Box 1982, Dammam 31441, Saudi Arabia; mmalthobaiti@iau.edu.sa

**Keywords:** functional near-infrared spectroscopy, optical biosensor, wearable brain sensing, brain–computer interface, melanin, skin tone, Monte Carlo, health equity, Fitzpatrick scale, wavelength optimization

## Abstract

Functional near-infrared spectroscopy (fNIRS) is an optical biosensing modality that transduces cortical hemodynamics into a measurable near-infrared signal, underpinning wearable brain monitors and brain–computer interfaces (BCIs). Because epidermal melanin is a strong near-infrared absorber, device sensitivity may depend on skin tone, raising an equity concern for optical neural biosensors. (1) Objective: We quantify, by Monte Carlo simulation, how epidermal melanin biases the cerebral sensitivity of continuous-wave fNIRS biosensors across the Fitzpatrick scale, and evaluate source-wavelength selection as a device-level mitigation. (2) Methods: Using a physically sourced melanin model and a two-stage design—an idealized layered slab for controlled sweeps and the Colin27 anatomical atlas for realistic-geometry confirmation—we computed gray-matter sensitivity for three skin tones, four wavelengths, and seven source–detector separations. (3) Results: Melanin acts as a surface gate that lowers absolute cerebral sensitivity by up to ~12.5-fold (at 690 nm) for dark skin—and by ~5-fold at the 850 nm optimum—while preserving depth selectivity; longer wavelengths reduce the penalty (dark-skin retained sensitivity rising from 8.0% at 690 nm to 29.6% at 940 nm) but never remove it; and 850 nm is a practical optimum for single-wavelength cerebral sensitivity, combining the highest absolute sensitivity with improved equity, whereas 940 nm gains relative equity only as absolute signal collapses through water absorption. Atlas simulations reproduce the 850 nm optimum in realistic anatomy under identical optical assumptions and show that idealized slabs overestimate sensitivity ~50-fold and that sensitivity varies ~19-fold with probe placement. (4) Conclusions: Skin tone and anatomy compound to disadvantage darker-skinned users of optical neural biosensors; wavelength selection and a sourced correction-factor framework offer concrete routes toward equitable device design.

## 1. Introduction

Functional near-infrared spectroscopy (fNIRS) is an optical biosensing modality that non-invasively transduces cortical hemodynamic activity into a measurable near-infrared signal [1]. As a wearable, motion-tolerant biosensor it has become central to brain–computer interfaces (BCIs), neuroergonomics, and rehabilitation, and continued instrumentation advances have produced increasingly compact and wearable optical neural sensors [2,3]. Our group and others have applied such systems to motor-cortex monitoring, BCI, and prosthetic control [3,4]. Optical neural interfaces such as fNIRS form part of the broader landscape of neural-interface technologies now being explored for applications well beyond healthcare [5].

For any wearable optical biosensor, signal quality depends on the light path between the device and the target tissue. In fNIRS this path begins at the epidermis, where melanin—a strong near-infrared absorber—is concentrated. Individuals with darker skin therefore attenuate the interrogating light more strongly before it reaches the cortex, and again on the return path to the detector. This physical dependence has a documented consequence: demographic under-reporting and phenotypic exclusion are common in fNIRS research, including the exclusion of participants with darker skin or coarser hair owing to poor signal quality [6]. A biosensor whose performance depends on skin tone is an equity problem as much as an engineering one.

Melanin’s optical effect has been characterized extensively for peripheral optical biosensors such as pulse oximeters and photoplethysmographic wearables, where Monte Carlo modeling and experiment have quantified skin-tone bias and motivated correction strategies [7,8,9,10,11,12]. These devices, however, interface with superficial tissue. Cerebral fNIRS is a far more demanding sensing problem: the light must additionally traverse scalp, skull, and cerebrospinal fluid to reach the cortex and return, so the melanin penalty compounds with depth. For this cerebral case the bias remains under-characterized. Prior simulation of optical neural interfaces has shown that penetration depends on wavelength and pigmentation and has flagged a patient-eligibility framework as future work, but has not quantified a cerebral sensitivity metric or a device-level mitigation [13].

Here we address this gap with a Monte Carlo characterization framed around biosensor design. We adopt cerebral sensitivity—the gray-matter contribution to the detected signal—as the figure of merit, because it captures how informative the biosensor is about the brain rather than raw attenuation. We pursue three questions relevant to equitable device design: (RQ1) how epidermal melanin biases cerebral sensitivity across the Fitzpatrick scale and source–detector separation; (RQ2) which source wavelength minimizes the skin-tone dependence; and (RQ3) whether a sourced correction factor can rescale sensitivity across skin tones, and what residual bias remains. A two-stage design—an idealized slab for controlled parametric sweeps and the Colin27 anatomical atlas [14] for realistic-geometry confirmation—keeps every optical input fixed to published sources so that no parameter is tuned to a desired outcome.

## 2. Materials and Methods

This study comprises two stages. Stage A uses a six-layer planar slab for a controlled parametric sweep over skin tone, wavelength, and source–detector separation (SDS), isolating the melanin effect from anatomical confounds. Stage B uses the Colin27 adult brain atlas [14] to confirm the principal trends in realistic, folded anatomy. All simulations are continuous-wave forward Monte Carlo computations of the fluence distribution; sensitivity is obtained by the forward–adjoint (source–detector) reciprocity method. No melanin parameter or tissue optical property was adjusted to obtain any reported result; all inputs were fixed a priori from the literature.

### 2.1. Monte Carlo Engine

Photon transport was simulated with a GPU-accelerated Monte Carlo package of the MCX family [15], implementing the voxel-based radiative-transport formalism of Wang, Jacques, and Zheng [16], on an NVIDIA GTX 1650 Super GPU. Each simulation launched 1 × 10^8^ photons unless otherwise stated; photon-count stability was confirmed by comparing 1 × 10^8^ and 1 × 10^9^ runs at representative conditions, for which the recovered gray-matter sensitivity changed by less than 3%. A fixed random seed and logged configuration files ensured reproducibility; the time window was 0–5 ns with a single gate, appropriate for continuous-wave sensing. Beyond photon-count convergence, the transport model was validated against analytical diffusion theory in a homogeneous medium (Section 2.7): the effective attenuation coefficient recovered from the Monte Carlo fluence agreed with the analytical value √(3μa(μa + μs′)) to within 0.4% (0.1733 vs. 0.1741 mm^−1^), and the fluence profile matched theory over more than four decades (RMS deviation 1.2%, R^2^ = 0.9999).

### 2.2. Melanin Optical Model

Epidermal absorption was modeled using the melanosome formulation compiled by Boonya-ananta et al. [10], following Jacques [17]. The melanosome absorption coefficient, the non-melanin baseline, and the mixed epidermal absorption are given by Equations (1)–(3):

(1) $$\mu_{a,mel}\left(\lambda \right)=6.6\times 10^{11}\cdot \lambda^{-3.33}\;\;\;\;\;\;\;\;\;\;\;\;\;[{cm}^{-1}]$$

(2) $$\mu_{a,0}\left(\lambda \right)=7.84\times 10^{8}\cdot \lambda^{-3.255}\;\;\;\;\;\;\;\;\;\;\;\;\;\left[{cm}^{-1}\right]$$

(3) $$\mu_{a,epi}\left(\lambda \right)=Mf\cdot \mu a,mel\left(\lambda \right)+\left(1-Mf\right)\cdot \mu a,0\left(\lambda \right)\left[{cm}^{-1}\right]$$

where λ is in nm and Mf is the melanosome volume fraction. The coefficient in Equation (1) was verified against the review’s tabulated anchor (evaluating at 523 nm reproduces the reported epidermal maximum) [10]. Fitzpatrick groups were assigned Mf = 0.04 (light, I–II), 0.13 (medium, III–IV), and 0.30 (dark, V–VI), representative mid-band values within the published ranges [10,17]. These three volume fractions are representative sample points along a continuous pigmentation axis rather than discrete optical categories; real skin pigmentation varies continuously, and the grouped values are used here to characterize the scale and wavelength dependence of the melanin effect.

### 2.3. Tissue Geometry and Optical Properties

The Stage A slab comprised, from the surface inward, a melanin-bearing epidermis (0.1 mm), dermis (3.9 mm), skull (6 mm), cerebrospinal fluid (CSF, 2 mm), gray matter (4 mm), and white matter (20 mm), at 0.1 mm isotropic resolution so the thin epidermis was resolved by one voxel. Deeper-layer properties at 760 and 850 nm were taken from our previously validated head model [18] (Table 1); scattering was specified as reduced scattering μ_s_′ and converted via μ_s_ = μ_s_′/(1 − g). Properties at 690 and 940 nm were obtained by anchored extrapolation: scattering using the Jacques power-law exponents [17] anchored to the published values, and absorption using the standard near-infrared water-absorption spectral shape [17]. This preserves the published anchors exactly while providing physically motivated 690/940 nm values; the water-absorption extrapolation is noted as a limitation (Section 5). The epidermis was assigned the scalp reduced-scattering value at each wavelength (9.18, 8.00, 7.50 and 6.50 mm^−1^ at 690, 760, 850 and 940 nm, respectively, and 7.44 mm^−1^ at 800 nm; see Table 1), with only its absorption made melanin-dependent. To reflect the near-isosbestic wavelength region used by commercial multi-wavelength fNIRS for oxygen-related recovery, an additional wavelength at 800 nm was included, with deeper-layer properties obtained by the same anchored-extrapolation procedure; its values are listed in Table 1.

### 2.4. Sensitivity Metric

For each source–detector pair, the spatial sensitivity was computed as the voxel-wise product of the forward (source) and adjoint (detector) fluence, the standard reciprocity construction of the measurement Jacobian. The primary metric, absolute gray-matter sensitivity Sgray, is the sum of this product over gray-matter voxels; the secondary metric, relative brain sensitivity RBS = Sgray/Stotal, quantifies depth selectivity. The melanin bias is expressed relative to the light-skin reference at matched wavelength and SDS. The common voxel volume is a constant factor that cancels in all reported ratios and in the correction factor, and sensitivities are normalized to the light-skin reference at matched wavelength and SDS. Physically, Sgray corresponds to the gray-matter-originating portion of the detected continuous-wave intensity—and hence of the modified Beer–Lambert optical-density change—so RBS expresses the fraction of a CW-fNIRS measurement that reflects cortical, rather than extracerebral, hemodynamics. Reported in arbitrary units proportional to detected signal per unit incident power, the curves may therefore be read on a per-mW basis. We note that this sensitivity is a raw-intensity quantity; a correction derived from it applies to mean sensitivity and is not, without further assumptions, a correction for recovered oxy-/deoxy-hemoglobin concentrations, which additionally depend on the wavelength pair and chromophore-recovery model. Sources and detectors were defined at surface tissue voxels in direct optical contact with the medium, with no air gap between the optode and the surface.

### 2.5. Array and Sweep

A multi-distance linear array was simulated with SDS of 8, 12, 16, 20, 24, 28, and 32 mm. The Stage A sweep spanned three skin tones × four wavelengths (690, 760, 850, 940 nm) × seven SDS (84 conditions), each evaluated by a forward field (reused across SDS) and per-SDS adjoint fields.

### 2.6. Stage B: Colin27 Atlas

Stage B used the Colin27 adult brain atlas [14] (181 × 217 × 181 voxels, 1 mm) segmented into scalp/soft tissue, skull, CSF, gray matter, white matter, and deep structures. Because the 1 mm resolution cannot represent a 0.1 mm melanin epidermis, melanin was characterized in Stage A (where the epidermis is resolved) and Stage B was used, without melanin, to quantify the effect of realistic anatomy. Optodes were placed on the scalp surface and directed into the head; sites were restricted to the upper convexity, where the surface normal is well-defined on the voxel grid. Two analyses were performed: a 15-site survey at 850 nm and 30 mm SDS to characterize the distribution and position-dependence of cerebral sensitivity, and a multi-wavelength survey repeating the identical 15 sites at 690, 760, 850, and 940 nm to test whether the Stage A wavelength optimum survives realistic geometry. A path-matched approach representing the epidermis as a thicker (1 mm) atlas layer with correspondingly scaled absorption was tested but rejected: within a 1 mm layer, scattering lengthens the photon path so that a volume-averaged absorption no longer reproduces the correct single-pass melanin attenuation (the dark/light sensitivity ratio deviated markedly from the resolved 0.1 mm slab). Melanin was therefore retained only in Stage A, and Stage B characterizes the effect of realistic anatomy without melanin; the combined pigmentation–anatomy effect is accordingly an inference drawn from the two analyses rather than a directly simulated result.

### 2.7. Benchmark Validation Against Diffusion Theory

To validate the physical accuracy of the transport model beyond photon-count convergence, the Monte Carlo solver was benchmarked against analytical diffusion theory in a large homogeneous medium. For a point source in such a medium, diffusion theory predicts a fluence rate φ(r) ∝ exp(−μeff·r)/r, with μeff = √(3μa(μa + μs′)). The effective attenuation coefficient recovered from the Monte Carlo fluence (from the slope of ln(φ·r) versus r) was 0.1733 mm^−1^, agreeing with the analytical value of 0.1741 mm^−1^ to within 0.4%, and the full fluence profile matched diffusion theory over more than four decades (root-mean-square deviation 1.2%; R^2^ = 0.9999). This confirms the model reproduces the correct transport physics; the remaining dependence on the assumed tissue optical properties is addressed in the Limitations.

## 3. Results

### 3.1. Melanin Bias Is a Surface Gate, Invariant Across Depth

Across all source–detector separations, the ratio of dark-skin to light-skin gray-matter sensitivity was essentially constant (Figure 1). At 760 nm, for example, dark skin retained 13.4% of the light-skin sensitivity at every SDS from 8 to 32 mm. Melanin thus acts as a surface gate, attenuating light entering and leaving the head irrespective of probing depth. Consistent with this, relative brain sensitivity (RBS) was nearly unchanged across skin tones (≈0.9–1.2% at 20 mm), confirming that melanin changes signal magnitude rather than depth selectivity. Absolute sensitivity, however, fell sharply with pigmentation: at 20 mm and 760 nm, dark-skin Sgray was 13.4% of the light-skin value, a ~7.5-fold reduction. Expressed as a loss factor, the reduction is largest at 690 nm (8.0% retention, a ~12.5-fold loss) and smallest at the 850 nm optimum (~5-fold), consistent with Figure 2 and Table 2.

### 3.2. Longer Wavelengths Reduce, but Do Not Eliminate, the Penalty

Dark-skin retained sensitivity increased monotonically with wavelength: 8.0% at 690 nm, 13.4% at 760 nm, 20.2% at 850 nm, and 29.6% at 940 nm (Figure 2), following the steep wavelength dependence of melanin absorption (μa ∝ λ^−3.33^). Even at 940 nm, however, dark skin retained less than one-third of the light-skin sensitivity, so wavelength selection mitigates but does not remove the bias.

### 3.3. 850 nm Is the Practical Optimum: The Melanin–Water Trade-Off

Although 940 nm minimized the relative skin-tone penalty, it produced the lowest absolute cerebral sensitivity of all wavelengths, because water absorption rising toward its ~970 nm peak strongly attenuates the deep signal (Figure 3). At 20 mm SDS, light-skin Sgray at 940 nm was roughly 60-fold lower than at 850 nm, so 940 nm’s superior equity is hollow. The 850 nm wavelength yielded the highest absolute sensitivity while also improving equity (20.2% retention), making it the practical optimum for an fNIRS biosensor. The optimal wavelength thus reflects a trade-off between melanin transmission (favoring longer wavelengths) and water absorption (favoring shorter wavelengths). This optimum refers to single-wavelength cerebral sensitivity among the wavelengths tested and under the stated optical assumptions; functional fNIRS additionally requires a wavelength pair chosen for oxy-/deoxy-hemoglobin recovery, considered in Section 4. The near-isosbestic 800 nm wavelength, added here, falls between 760 and 850 nm on both the equity and absolute-sensitivity axes (Figure 3) and yields a dark-skin correction factor of 6.0× (16.8% retention; Table 2).

### 3.4. A Sourced Correction-Factor Characterization

Because the penalty is nearly independent of SDS (Section 3.1), the deficit can be captured by a single multiplicative correction factor C = Sgray(light)/Sgray(tone) per skin tone and wavelength (Table 2, Figure 4), decreasing from 12.5× (dark, 690 nm) to 3.4× (dark, 940 nm). We emphasize that C is a bias-correction, not a signal-to-noise remedy: rescaling a dark-skin measurement upward by 5–12× also amplifies its noise, so the correction restores comparability of mean sensitivity but does not recover lost photons—an important caveat for any correction built on it. We present these factors as an illustrative, first-order characterization of the magnitude and wavelength dependence of the melanin bias, not as a ready device-calibration prescription: they capture the melanin contribution only, and not epidermal-thickness variation, scattering differences, optode contact pressure and coupling, hair, or individual extracerebral anatomy, all of which additionally affect attenuation.

### 3.5. Realistic Anatomy Greatly Reduces Cerebral Sensitivity (Stage B)

Across 15 probe placements on the Colin27 convexity (850 nm, 30 mm SDS), median RBS was 0.09%, versus 4.6% for the idealized slab—an approximately 50-fold overestimation by the slab (Figure 5). Even the best atlas site (0.42%) was ~11-fold below the slab. Figure 6 visualizes this: the measurement sensitivity is largely confined to scalp and skull, with only faint sensitivity reaching the folded cortex.

**Figure 5:**
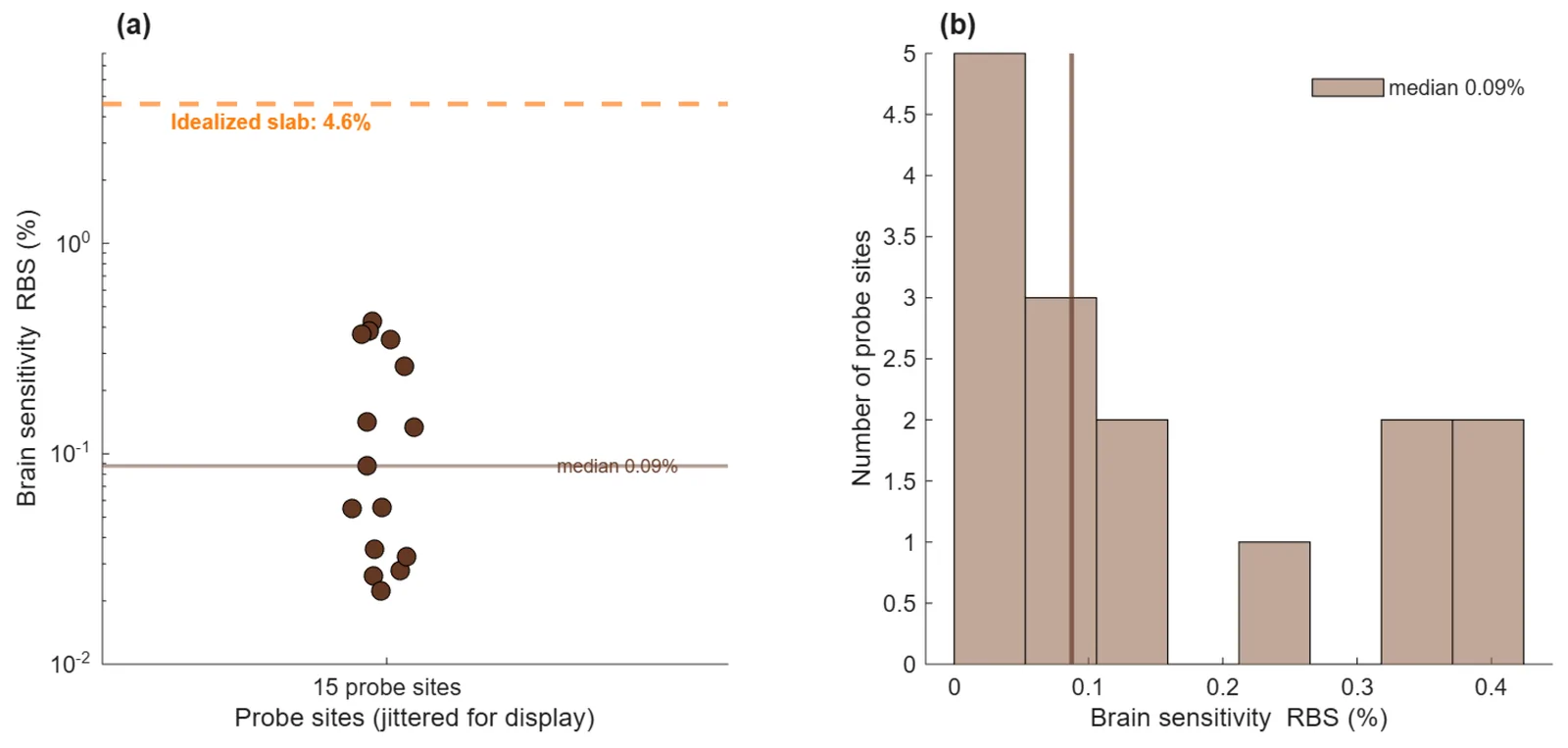
Distribution of cerebral sensitivity (RBS) across 15 probe placements on the Colin27 convexity (850 nm, 30 mm SDS; Stage B). **Left**: Per-site values with median against the idealized-slab value. **Right**: Histogram. Real-anatomy sensitivity is far below the slab prediction and varies widely across sites. (**a**) Left panel: Per-site relative brain sensitivity RBS (%, vertical axis) for the 15 probe sites (horizontal axis: probe-site index); the dashed line marks the idealized-slab value. (**b**) Right panel: Histogram of the same RBS values (horizontal axis: RBS in %; vertical axis: number of sites). Panels are referred to as Figure 5a,b in the text. This distribution is shown at 850 nm, the optimum identified in Figure 3 and Figure 7.

**Figure 6:**
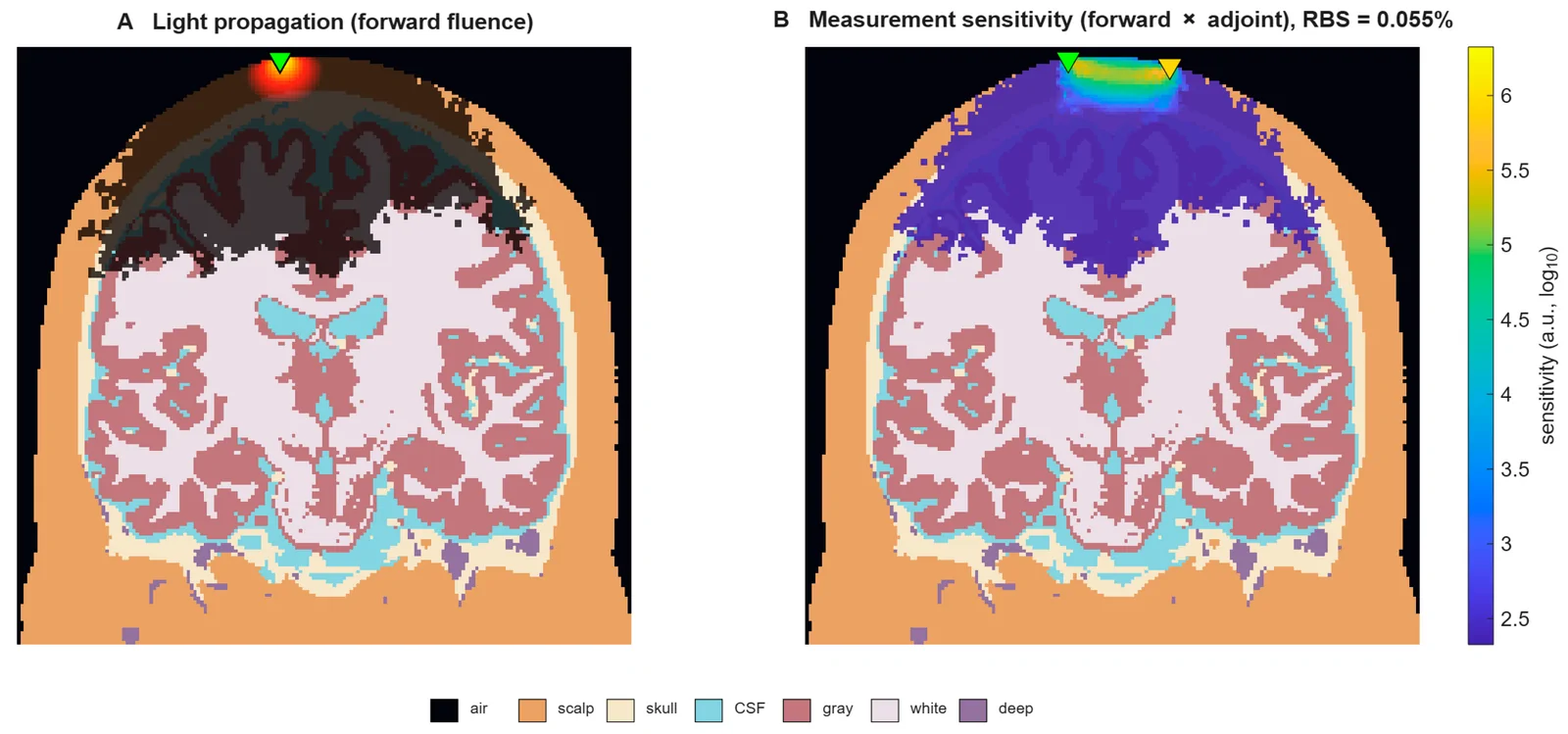
Photon sensitivity over real cortical anatomy (Colin27 cross-section, 850 nm, 30 mm SDS; Stage B). (**A**) Forward fluence from the source, concentrated in superficial tissue. (**B**) Measurement sensitivity (forward × adjoint), which reaches the folded gray matter only weakly (RBS = 0.055% at this site). The spatial sensitivity map is shown at 850 nm, the optimum wavelength.

**Figure 7:**
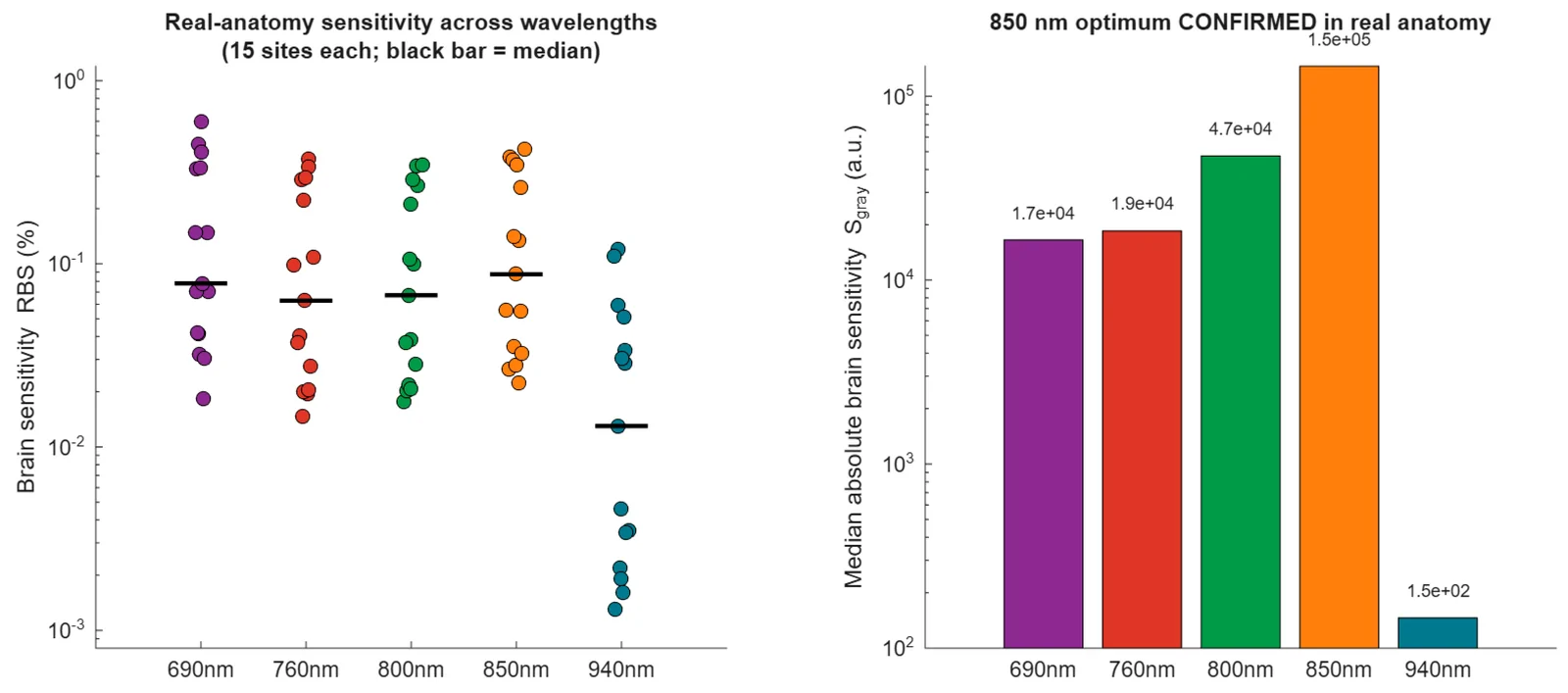
Multi-wavelength atlas survey (Stage B). **Left**: per-site cerebral sensitivity (RBS) at 690, 760, 800, 850, and 940 nm across the identical 15 sites. **Right**: median absolute cerebral sensitivity by wavelength; 850 nm is highest and 940 nm collapses, reproducing the Stage A optimum in realistic anatomy.

### 3.6. Cerebral Sensitivity Is Strongly Position-Dependent (Stage B)

RBS varied approximately 19-fold across the 15 sites (0.02–0.42%; Figure 5), driven by local gyral geometry and extracerebral thickness. Biosensor cerebral sensitivity is therefore highly dependent on precise probe placement relative to cortical folds—variability the slab cannot capture.

### 3.7. The 850 nm Optimum Is Reproduced in Realistic Anatomy (Stage B)

Repeating the survey at all five wavelengths reproduced the Stage A ordering in real anatomy (Figure 7): median absolute cerebral sensitivity was highest at 850 nm (1.46 × 10^5^ a.u.) and collapsed at 940 nm (1.46 × 10^2^ a.u.), independently reproducing the melanin–water trade-off. This convergence of two independent geometries on the same 850 nm optimum strengthens the wavelength recommendation for device design. Because both stages use the same anchored 940 nm absorption extrapolation, this agreement demonstrates internal consistency and reproduction of the wavelength ordering under identical assumptions; it does not independently validate the 940 nm absorption model, which remains contingent on that extrapolation. The transport model underlying all of these results was independently validated against analytical diffusion theory (Section 2.7, Figure 8). The added 800 nm wavelength yields a median gray-matter sensitivity of 4.7 × 10^4^ a.u., between the 760 and 850 nm values, and 850 nm remains the atlas optimum (Figure 7).

## 4. Discussion

These simulations quantify, for cerebral optical biosensing, an equity concern previously documented mainly at the empirical level [6]. The central result is that epidermal melanin does not change where fNIRS samples—depth selectivity is preserved—but how much cortical signal survives, collapsing absolute cerebral sensitivity by up to ~12.5-fold (at 690 nm) for dark skin. This matches the clinical observation that skin-tone effects in fNIRS manifest as elevated channel rejection and reduced signal quality rather than distorted depth profiles, and it identifies the epidermis as the biosensor’s limiting interface.

Source-wavelength selection emerges as a simple, algorithm-independent design lever, and our identification of 850 nm as the practical optimum is directly actionable for optode and source selection in wearable fNIRS devices. The analysis also cautions against assuming longer is always better: the melanin–water trade-off means 940 nm, despite the best relative equity, yields the weakest absolute signal—an effect reproduced independently in realistic anatomy. Complementary strategies address other confounds and objectives of optical head measurements, including the influence of hair [19], the optimization of photon delivery to cortical targets [20], and the integration of fNIRS with EEG in assistive and prosthetic systems [21].

Because functional fNIRS recovers oxy- and deoxy-hemoglobin changes from attenuation at two or more wavelengths, the single-wavelength optimum must be extended to wavelength-pair selection. Using standard hemoglobin molar extinction coefficients, the separability of Δ[HbO] and Δ[HbR]—and the associated crosstalk—is governed by the conditioning of the 2 × 2 extinction matrix, quantified by its condition number κ. The pairs best conditioned for chromophore separation are anchored at 690 nm (e.g., 690 + 940 nm, κ = 2.27; 690 + 850 nm, κ = 2.58), yet these are the least equitable, incurring the full ~12.5-fold melanin penalty at 690 nm. Conversely, the near-isosbestic 800 + 850 nm pairing standard for oximetry is comparatively ill-conditioned for separation (κ = 11.7), because both wavelengths lie on the same side of the isosbestic point. The pairs 760 + 850 nm (κ = 3.21) and 760 + 940 nm (κ = 2.83) offer the best joint compromise, remaining well-conditioned while retaining substantially better dark-skin sensitivity (13.4%) than any 690 nm pairing. The melanin penalty and the separability optimum thus pull in opposite directions in wavelength space, and an equitable multi-wavelength design must weigh both. A full quantitative recovery analysis for specified physiological Δ[HbO]/Δ[HbR] changes additionally requires a realistic instrument-noise model, differential pathlength factors, input optical power and detector responsivity, and is identified here as the clearly motivated next stage of this work. Beyond hemodynamic oxygenation, the same Monte Carlo sensitivity framework could be extended to cerebral blood-flow measurement—for example diffuse correlation spectroscopy and related deep-tissue flow techniques—where achieving adequate brain sensitivity through pigmented extracerebral tissue is likewise a central challenge [22]; melanin-related bias and its wavelength dependence therefore merit dedicated study in that modality as well.

A practical consequence concerns calibration by input power. The melanin deficit cannot, in general, be offset simply by increasing source power: continuous skin irradiation in the 700–1050 nm range is limited to a maximum permissible exposure of 0.30 W cm^−2^ (ANSI Z136.3), and skin-contact devices are further bound by temperature limits (IEC 60601-1). A 12.5-fold dark-skin deficit at 690 nm therefore cannot be recovered by a 12.5-fold power increase without exceeding the skin-safety and heating limits, reinforcing that the correction factor addresses bias in mean sensitivity rather than the underlying signal-to-noise and safety constraints.

The correction-factor framework offers a sourced means of comparing sensitivity across skin tones, but its honest interpretation is essential: it corrects bias in mean sensitivity, not the signal-to-noise deficit, and cannot recover undetected photons. It is therefore a tool for quantitative comparability and equitable study design, not a cure for the underlying inequity.

The atlas results place these findings in sobering context. Realistic anatomy already renders cerebral sensitivity marginal—about 50-fold below idealized-slab predictions—and highly position-dependent. The equity implication compounds: a user with darker skin (retaining at best ~20% of light-skin sensitivity at 850 nm) and less favorable local anatomy faces the largest combined deficit. This intersection of pigmentation and anatomy is, to our knowledge, a novel quantification and a concrete target for equitable optical-biosensor design—through wavelength selection, array optimization, and transparent reporting of expected sensitivity ranges across populations. The results complement peripheral-tissue melanin studies [7,8,9,10,11,12], and related analyses of skin optical properties and of pigmentation effects in tissue oximetry [23,24], by extending the analysis to the cerebral case and build on our group’s prior Monte Carlo modeling of skin-color effects in near-infrared sensing [25].

### Limitations

Several limitations qualify these results. The correction factor is a bias-correction, not a signal-to-noise remedy. The 940 nm results depend on the anchored water-absorption extrapolation used for deeper-layer absorption; although the melanin–water trade-off is physically well established and reproduced across both stages, the magnitude of the 940 nm collapse is contingent on this extrapolation. In Stage B the atlas ‘scalp’ label represents combined extracerebral soft tissue (~11–15 mm) assigned scalp optical properties, so atlas absolute sensitivities are indicative; the robust findings are the relative gap from the slab and the cross-site variability. Voxel-grid optode placement on curved anatomy is approximate, and sites were restricted to the upper convexity; a full, clinically indexed cortex-wide characterization would benefit from mesh-based Monte Carlo and is left to future work. Finally, this study is entirely in silico and does not constitute clinical validation; extension to recovered chromophore accuracy and experimental confirmation are natural next steps. Throughout, equity is defined operationally as the retained cerebral sensitivity of darker skin expressed as a percentage of the light-skin value at matched wavelength and SDS (equivalently, the reciprocal of the melanin correction factor). A full instrument-level analysis of chromophore-recovery accuracy and of which channels fall below the noise-variance detection threshold for a given input power was outside the present transport-only scope, as it requires realistic noise, pathlength, power and detector models together with the skin-exposure limits noted above; this is identified as the next stage of the work.

## 5. Conclusions

Using physically sourced Monte Carlo simulations across the Fitzpatrick scale, we showed that epidermal melanin acts as a surface gate that reduces absolute cerebral fNIRS sensitivity by up to ~12.5-fold (at 690 nm) for dark skin while preserving depth selectivity. Longer wavelengths reduce but cannot eliminate this penalty, and 850 nm emerges as a practical optimum balancing cerebral signal strength against skin-tone equity—reproduced independently in realistic Colin27 anatomy under identical optical assumptions. Realistic anatomy further reveals that idealized slabs overestimate cerebral sensitivity ~50-fold and that sensitivity varies ~19-fold with probe placement, so darker skin combined with unfavorable anatomy produces the largest compounded deficit. We provide a sourced correction-factor framework to support equitable optical-biosensor design, while cautioning that it addresses bias rather than signal-to-noise. These results offer a quantitative foundation and concrete design guidance for making cerebral fNIRS biosensors more equitable across skin tones.

## Figures and Tables

**Figure 1:**
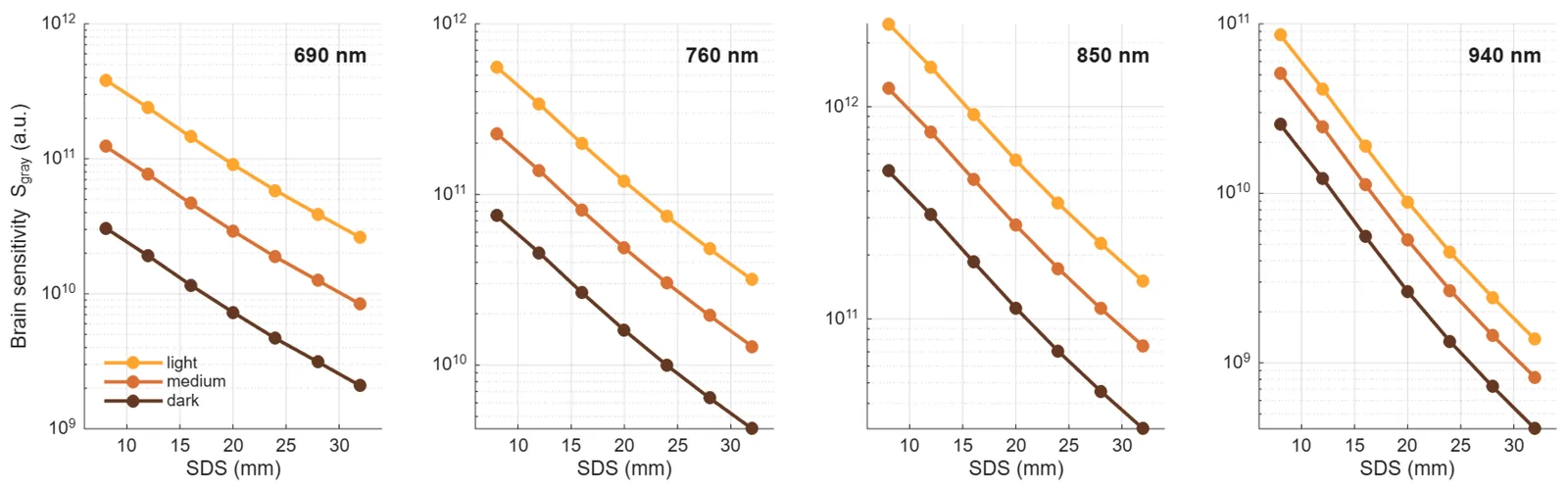
Absolute cerebral sensitivity (Sgray) versus source–detector separation for light, medium, and dark skin at 690, 760, 850, and 940 nm (Stage A slab). The dark-to-light ratio is approximately constant across separation at each wavelength, indicating a surface-gating mechanism. Vertical axis logarithmic (arbitrary units).

**Figure 2:**
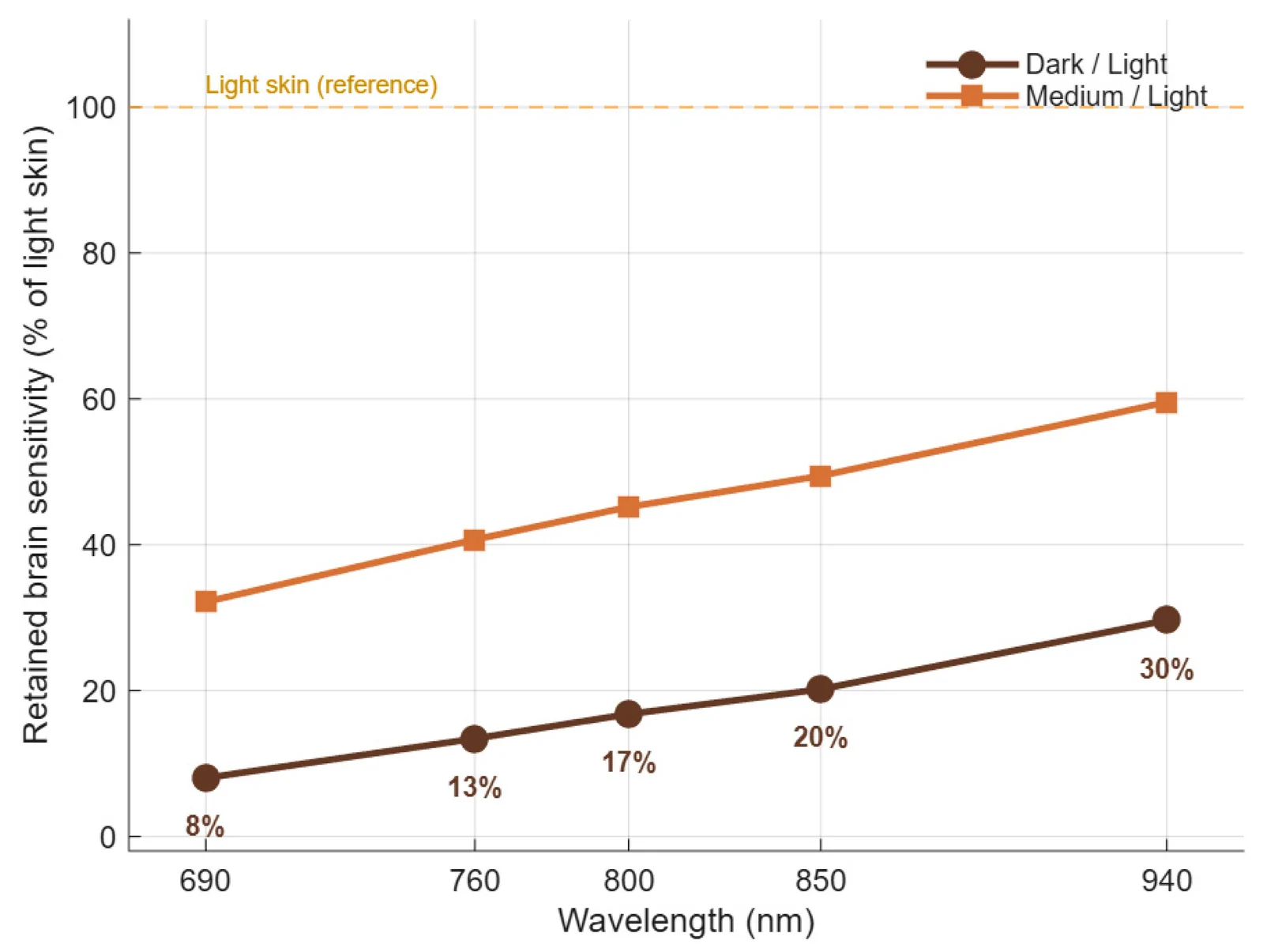
Retained cerebral sensitivity of medium and dark skin, as a percentage of the light-skin value, versus wavelength (Stage A). The penalty decreases at longer wavelengths but remains well below parity at all wavelengths.

**Figure 3:**
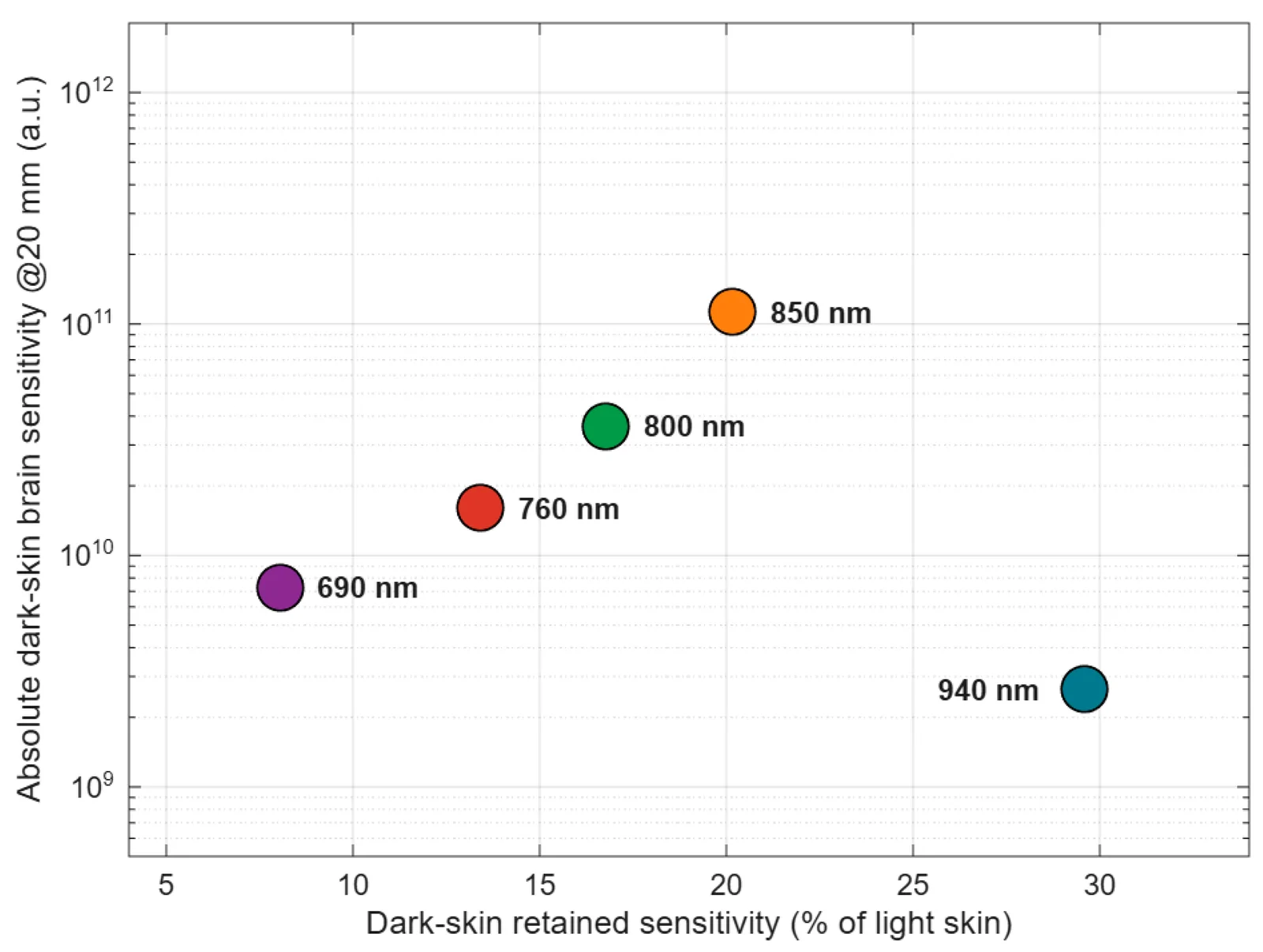
The melanin–water trade-off (Stage A). Each wavelength is plotted by skin-tone equity (dark-skin retention) against absolute dark-skin cerebral sensitivity at 20 mm SDS (logarithmic). The 850 nm wavelength occupies the favorable region; 940 nm achieves the best equity but the lowest absolute signal. The near-isosbestic 800 nm wavelength is now included (between 760 and 850 nm).

**Figure 4:**
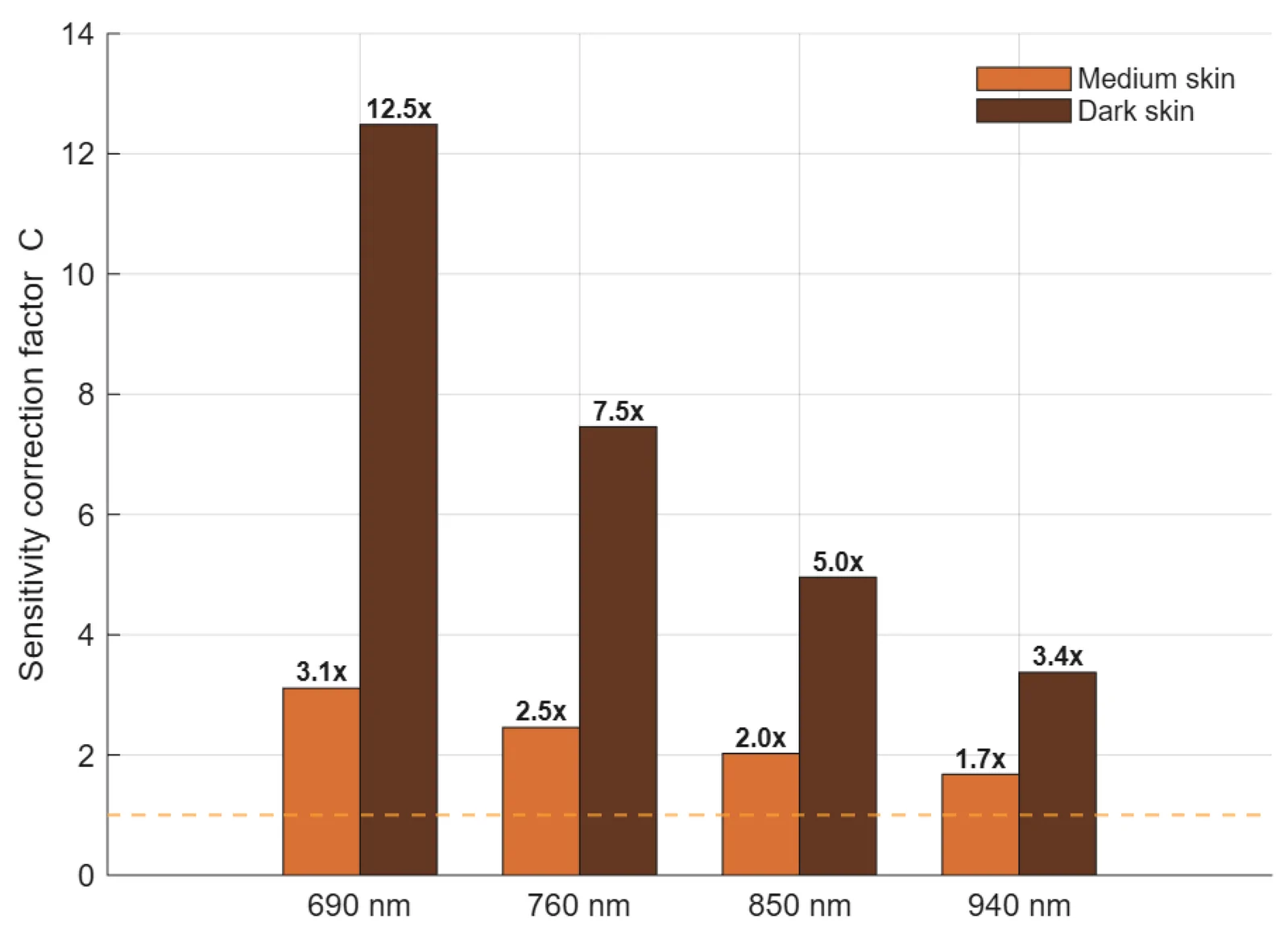
Melanin correction factor by wavelength for medium and dark skin (Stage A). Bars show the sensitivity scale-up required to match light-skin sensitivity; the factor is largest at short wavelengths and for dark skin.

**Figure 8:**
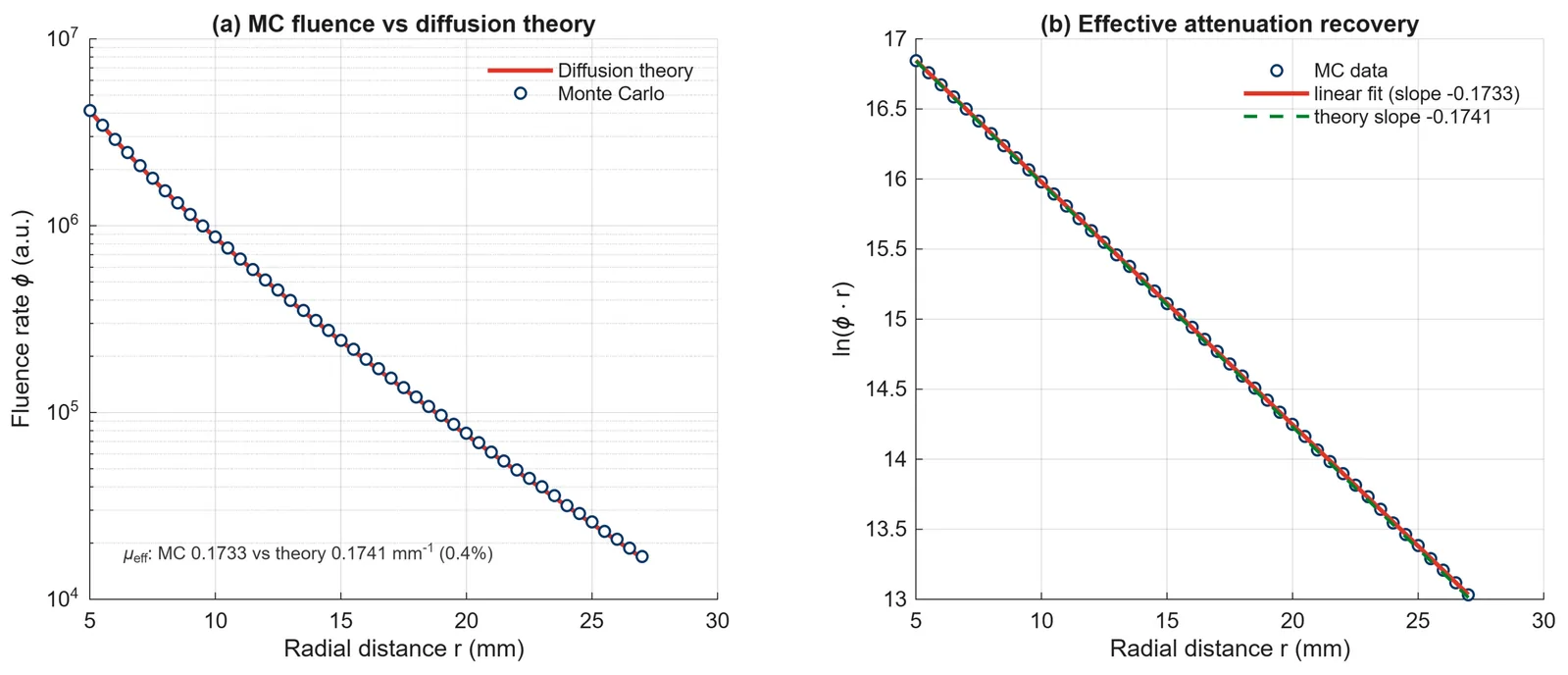
Benchmark validation of the Monte Carlo model against analytical diffusion theory in a homogeneous medium. (**a**) Monte Carlo fluence rate φ (points) versus radial distance r, with the analytical diffusion prediction (line); agreement spans more than four decades. (**b**) Linearized form ln(φ·r) versus r; the fitted slope gives the effective attenuation coefficient μeff = 0.1733 mm^−1^, matching the analytical √(3μa(μa + μs′)) = 0.1741 mm^−1^ to within 0.4% (RMS deviation 1.2%, R^2^ = 0.9999).

**Table 1:** Frozen optical properties used in the simulations. Values at 760 and 850 nm for deeper layers are from the validated head model [18]; 690 and 940 nm values are anchored extrapolations (Section 2.3). Epidermal absorption follows Equations (1)–(3). μ_a_ and μ_s_′ in mm^−1^. The table also includes the epidermal reduced-scattering values and an additional column at 800 nm (the near-isosbestic wavelength); 800 nm deeper-layer values were obtained by the same anchored-extrapolation procedure as 690 and 940 nm.

Tissue	Property	690 nm	760 nm	800 nm	850 nm	940 nm	g	n
Epidermis (light)	μ_a_	0.973	0.705	0.595	0.486	0.348	0.89	1.37
Epidermis (dark)	μ_a_	7.003	5.076	4.279	3.497	2.501	0.89	1.37
Scalp/Dermis	μ_a_	0.017	0.018	0.0176	0.016	0.031	0.89	1.37
Scalp/Dermis	μ_s_′	9.18	8.00	7.48	7.50	6.50	—	—
Skull	μ_a_	0.015	0.016	0.0158	0.014	0.021	0.89	1.37
Skull	μ_s_′	10.53	10.00	9.78	9.50	9.00	—	—
CSF	μ_a_	0.002	0.004	0.0034	0.004	0.026	0.89	1.37
CSF	μ_s_′	0.01	0.01	0.01	0.01	0.01	—	—
Gray matter	μ_a_	0.020	0.022	0.0215	0.018	0.036	0.89	1.37
Gray matter	μ_s_′	11.68	10.00	9.78	9.50	8.08	—	—
White matter	μ_a_	0.024	0.025	0.0246	0.022	0.038	0.84	1.37
White matter	μ_s_′	14.02	12.00	11.56	11.00	9.35	—	—

**Table 2:** Melanin sensitivity correction factor C = Sgray(light)/Sgray(tone), by skin tone and wavelength (Stage A). The factor is nearly independent of source–detector separation. Dark-skin retained sensitivity (% of light) is shown for reference.

Wavelength	Medium C	Dark C	Dark Retention (%)
690 nm	3.1×	12.5×	8.0
760 nm	2.5×	7.5×	13.4
800 nm	2.2×	6.0×	16.8
850 nm	2.0×	5.0×	20.2
940 nm	1.7×	3.4×	29.6

## Data Availability

The Monte Carlo configuration files, frozen optical-property tables, and derived sensitivity datasets are available from the author upon reasonable request. The Colin27 adult brain atlas is publicly available.
